# Relevance of Distinct Monocyte Subsets to Clinical Course of Ischemic Stroke Patients

**DOI:** 10.1371/journal.pone.0069409

**Published:** 2013-08-02

**Authors:** Muichi Kaito, Shin-Ichi Araya, Yuichiro Gondo, Michiyo Fujita, Naomi Minato, Megumi Nakanishi, Makoto Matsui

**Affiliations:** Department of Neurology, Kanazawa Medical University, Uchinada, Ishikawa Prefecture, Japan; Tokyo Metropolitan Institute of Medical Science, Japan

## Abstract

**Background and Purpose:**

The most common strategy for treating patients with acute ischemic stroke is thrombolytic therapy, though only a few patients receive benefits because of the narrow time window. Inflammation occurring in the central nervous system (CNS) in association with ischemia is caused by immune cells including monocytes and involved in lesion expansion. If the specific roles of monocyte subsets in stroke can be revealed, they may become an effective target for new treatment strategies.

**Methods:**

We performed immunological examinations of 36 consecutive ischemic stroke patients within 2 days of onset and compared the results with 24 age-matched patients with degenerative disorders. The stroke patients were repeatedly tested for the proportions of monocyte subsets in blood, and serum levels of pro- and anti-inflammatory cytokines immediately after admission, on days 3-7 and 12-16 after stroke onset, and on the day of discharge. In addition, immunological measurements were analyzed for relationships to stroke subtypes and complications, including progressive infarction (PI) and stroke-associated infection (SAI).

**Results:**

Monocyte count was significantly increased from 0–16 days after stroke as compared to the controls (p<0.05). CD14^high^CD16^-^ classical and CD14^high^CD16^+^ intermediate monocytes were significantly increased from 0-7 and 3-16 days after stroke, respectively (p<0.05), whereas CD14 ^dim^CD16^high^ non-classical monocytes were decreased from 0–7 days (p<0.05). Cardioembolic infarction was associated with a persistent increase in intermediate monocytes. Furthermore, intermediate monocytes were significantly increased in patients with PI (p<0.05), while non-classical monocytes were decreased in those with SAI (p<0.05). IL-17A levels were positively correlated with monocyte count (r=0.485, p=0.012) as well as the percentage of non-classical monocytes (r=0.423, p=0.028), and negatively with that of classical monocytes (r=-0.51, p=0.007) during days 12-16.

**Conclusions:**

Our findings suggest that CD14^high^CD16^+^ intermediate monocytes have a role in CNS tissue damage during acute and subacute phases in ischemic stroke especially in relation to cardioembolism.

## Introduction

Acute ischemic stroke disturbs the neuronal circuitry and disrupts the blood brain barrier (BBB), which can lead to functional disabilities in daily life, with thrombolytic therapy such as with a tissue plasminogen activator commonly used for affected individuals. However, only a minority of patients receive this therapy because of the narrow time window and there is a large need for a treatment that can be utilized more than 4.5 hours after stroke onset, showing the necessity to develop new strategies to treat stroke patients [1].

Immunity and inflammation play pivotal roles in the pathogenesis of acute stroke. Various elements of the immune system are involved in all stages of the ischemic cascade, from acute intravascular events to parenchymal processes, leading to brain damage and tissue repair [2]. For example, expressions of various cytokines at the site of ischemia have been reported, including interleukin (IL)-1, -2, -3, -4, -6, -8, and -10, tumor necrosis factor (TNF)-α, and interferon, as well as numerous chemokines and growth factors [3]. In the periphery after stroke, systemic activation of the immune system occurs with increased levels of cytokines such as IL-1β [3], IL-6 [4,5], TNF-α [4], IL-10 [5], IL-17 [6], and transforming growth factor (TGF)-ß [5]. In addition, previous studies have suggested that an influx of different immune cells, including T cells, B cells, monocytes, and dendritic cells, as well as cytokines produced in the brain can exert immunomodulatory effects on post-ischemic inflammation [2,7,8].

There is broad consensus that stroke can lead to immunosuppression in the immediate stage, increasing the risk of infection and also likely contributing to prevent development of subsequent brain damage [9–11]. Also, several studies have shown that monocytes, which are circulating antigen-presenting leukocytes, play crucial roles in inflammation, T-cell differentiation, phagocytosis, and innate immunity [12]. Experimental data have shown that peripheral monocytes acutely recruited to the infarct area within the first 24 hours of tissue ischemia originate from a splenic reservoir and may be beneficial for mitigating stroke-induced brain injury [13]. Furthermore, rapid and prolonged functional deactivation of monocytes has been observed after acute stroke in humans, which is characterized by reduced expression of human leukocyte antigen (HLA)-DR and low *ex vivo* production of TNF-α [14,15].

Recent studies have reported that monocyte subpopulations in humans can be defined based on expressions of the lipopolysaccharide receptor CD14 and immunoglobulin Fc-gamma receptor-3 CD16 [16]. In normal individuals, 80-90% of circulating monocytes express CD14^high^CD16^-^ and are usually referred to as classical monocytes, which express high levels of the chemokine receptor CCR2 and low levels of CX3CR1 [17]. The remaining 5-10% possess CD16 molecule, and express high levels of CX3CR1 and low levels of CCR2 [17]. CD16^+^ monocytes consist of two subsets, one with low expression of CD14 (CD14 ^dim^CD16^+^) and the other with high expression of CD14 (CD14^high^CD16^+^), which are designated as non-classical and intermediate monocytes, respectively [16].

There may be specialized roles for those monocyte subsets in stroke pathophysiology and they might become targets for therapeutic intervention if their mechanisms are more precisely elucidated. We investigated the dynamics of monocyte subsets in blood of acute patients with ischemic stroke, and found that the percentages of CD14^high^CD16^-^ classical monocytes (classical Mo) and CD14^high^CD16^+^ intermediate monocytes (intermediate Mo) were increased, whereas that of CD14 ^dim^CD16^high^ non-classical monocytes (non-classical Mo) was decreased after stroke. Furthermore, intermediate Mo appeared to be involved in CNS tissue damage, while non-classical Mo were relevant to stroke-associated infection.

## Materials and Methods

### Ethics statement

The study protocol was approved by the institutional ethics committee of Kanazawa Medical University. All patients or their legal representatives gave written informed consent.

### Subjects

Between August 2010 and July 2011, we prospectively studied 36 consecutive patients with ischemic stroke hospitalized within 2 days after onset, who were previously independent in their activities of daily living. As disease controls, we enrolled 24 age-matched non-stroke patients who were admitted to our hospital during the same period and afflicted with other non-inflammatory neurological diseases, including Parkinson’s disease, multiple system atrophy, corticobasal degeneration, motor neuron disease, idiopathic normal pressure hydrocephalus, and cervical spondylosis.

Exclusion criteria were transient ischemic attack (defined using standard criteria), concurrent infection, tumor bearing state, history of chronic inflammatory disease, use of drugs such as antibiotics, immunosuppressants, and steroids within the preceding 3 months, and declining to participate.

Clinical diagnosis of ischemic stroke was confirmed by computed tomography (CT) or magnetic resonance (MR) imaging, with cranial CT/MR scans performed when neurological deterioration raised suspicion of worsening infarction including hemorrhagic transformation or new ischemic events. Blood samples were obtained from individual patients immediately on admission before medication was given (day 0-2 after onset; median 1.0, hyperacute phase), and then between 06:30 and 09:00 on day 3-7 (median 4.0, acute phase) and day 12-16 (median 14.0, early subacute phase), and finally on the day of hospital discharge (median 46.0, late subacute/chronic phase). Blood samples were obtained once from control patients after obtaining informed consent. Neurologic impairment was assessed daily using the National Institutes of Health Stroke Scale (NIHSS) and functional impairment was assessed with the modified Ranking Scale (mRS). Fixed members of a stroke team were engaged in treatment of all patients in the neurological intensive care unit according to the Japanese Guidelines for the Management of Stroke presented in 2009 [18].

We focused on early neurological deterioration in acute cerebral infarction, which was defined as an increase in NIHSS score by 2 or more points during the first week according to the European Progressing Stroke Study [19] and designated as progressing infarction (PI). According to the ESPIAS trial [20], patients with stroke-associated infection (SAI) were defined using the following criteria; body temperature >37.8^°^C along with the presence of suggestive clinical symptoms (productive cough, dyspnea, pleuritic pain, urinary tract symptoms), or blood leukocyte count >11.0×10^9^/L or <4.0×10^9^/L, pulmonary infiltration in chest X-ray findings, or cultures positive for a pathogen, which became overt within the first 7 days of stroke onset. We classified stroke etiology into 5 categories according to the TOAST criteria [21], which were large artery atherosclerosis (LAA), cardioembolism (CE), small artery occlusion (SAO), stroke of other determined cause (SOC), and stroke of undetermined cause (SUC).

### Neuro-imaging

CT images were acquired using a multislice CT scanner (Somatom-16, Siemens Medical Systems). MR images were acquired with a 1.5 or 3 tesla scanner (MR-Symphony or MR-TRIO, respectively, Siemens Medical Systems).

### Definition of monocyte subsets

**Figure 1:**
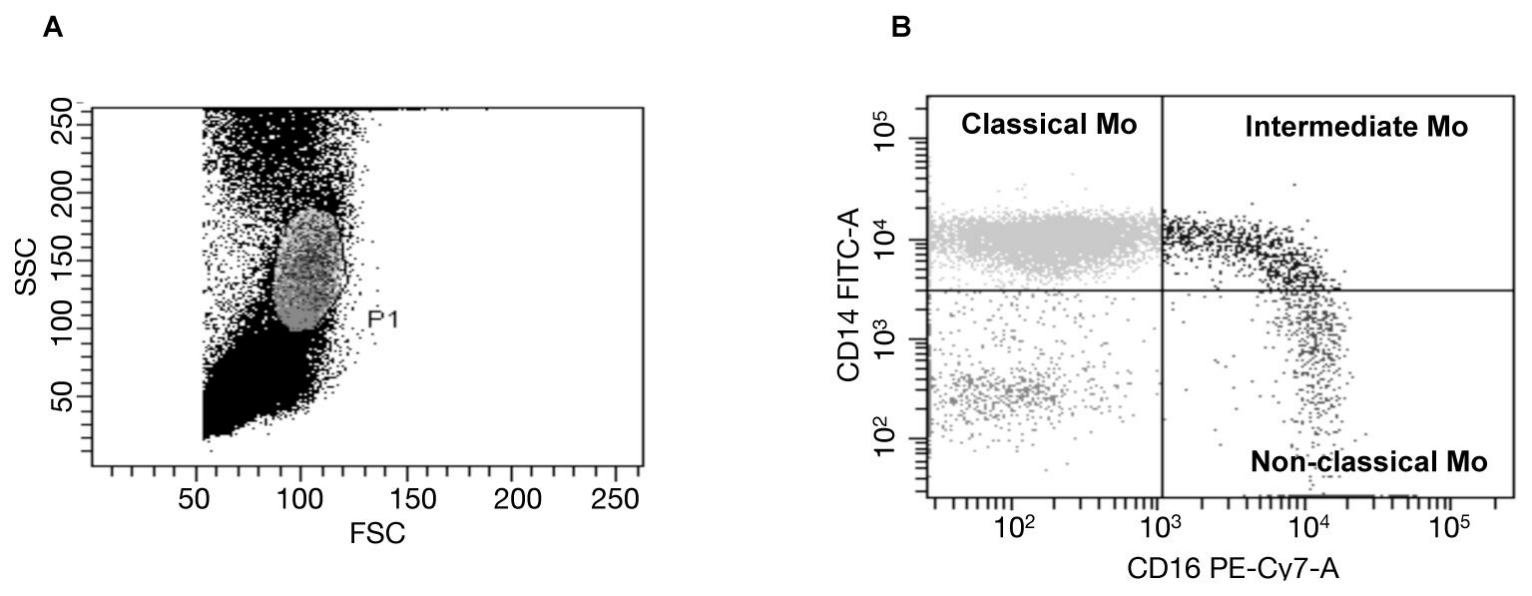
Analysis of monocyte subsets. (**A**) Monocytes were gated in a circle using forward scatter (FSC) and side scatter (SSC) plots. (**B**) Classical monocytes were identified by high expression of CD14 and no expression of CD16 (CD14^high^CD16^-^), intermediate monocytes by high expression of CD14 and various levels of positivity for the CD16 molecule (CD14^high^CD16^+^), and non-classical monocytes by scant expression of CD14 and high expression of CD16 (CD14 ^dim^CD16^high^). The proportions of each subset were evaluated by comparing the number of cells (dots) in the individual compartment to the total number of monocytes enclosed in the circle designated as P1.

Ethylenediaminetetraacetic acid (EDTA)-anticoagulated venous blood samples were collected from the subjects prior to each analysis. After separating mononuclear cells, monocytes were stained using fluorescein isothiocyanate (FITC)-labelled anti-CD16 and phycoerythrin/cyanin 7 (PE-Cy7)-conjugated anti-CD14 monoclonal antibodies (BD Biosciences, San Jose, USA), then analyzed with a BD FACSCanto flow cytometer (BD Biosciences, San Jose, USA) (Figure 1). Determination of CD14 and CD16 positivity was made using the appropriate isotype control antibody in keeping with the current consensus [16]. Monocyte subsets were defined as CD14^high^CD16^-^ classical, CD14^high^CD16^+^ intermediate, and CD14 ^dim^CD16^high^ non-classical monocytes [16].

### Measurement of cytokines

Levels of IL-1β, IL-6, IL-10, IL-17A, TNF-α, and TGF-β concentrations in serum samples obtained from stroke patients during 0-2, 3-7, and 12-16 days after stroke onset, as well as on the day of discharge were measured using cytometric bead array technology. The FACSCanto flow cytometer was used for data acquisition, with FCAP Array v 2.02 software (Burnsville, Minnesota, USA) utilized for data analysis. All procedures were done by one of the investigators blinded to the clinical and radiological data. The assay sensitivity reported by the manufacturer (BD Biosciences, San Diego, USA) was 2.3 ng/L for IL-1β, 1.6 ng/L for IL-6, 0.13 ng/L for IL-10, 0.3 ng/L for IL-17A, 1.2 ng/L for TNF-α, and 14.9 ng/L for TGF-β.

### Statistical analysis

Differences in numerical values were analyzed using Student’s t-test, Fisher exact test, Mann–Whitney’s U-test, a paired t-test, and analysis of variance (ANOVA), as appropriate. The time course of monocyte subsets was assessed with a paired t-test or ANOVA, as appropriate. Correlations were analyzed using Pearson’s correlation or Spearman’s rank correlation coefficient. All tests were performed using StatMate III (version 14.0). Probability values less than 0.05 were considered to be statistically significant.

## Results

### Clinical courses of patients

The demographic characteristics of the 36 patients with ischemic stroke and 24 with other neurological diseases are summarized in Table 1. There were no significant differences for age or gender, whereas hypertension, diabetes mellitus, atrial fibrillation, and chronic heart failure were significantly more frequent in stroke as compared to the control patients. The clinical courses and complications of the stroke patients are summarized in Table 2. PI including hemorrhagic transformation (n=3) occurred in 13 (36.1%) patients within 7 days after stroke onset, while SAI including pneumonia (n=8) and urinary tract infection (n=1) occurred in 9 (25.0%) during the same period. Most of those complications were observed with stroke due to LAA (7 of 14 patients, 50%) and CE (6 of 10, 60%). In contrast, only 2 (20%) with stroke due to SAO were complicated with PI.

**Table 1:** Demographic characteristics of stroke and control groups.

	Stroke patients	Control patients	p value
Number	36	24	
Age	68.6±11.9	66.9±6.6	NS
Gender (F:M)	18:18	11:13	NS
Risk factors			
Smoking	15 (41.7)	7 (29.2)	NS
Hypertension	28 (77.8)	11 (41.2)	0.011*
Diabetes mellitus	17 (47.2)	3 (12.5)	0.012*
Dyslipidemia	16 (44.4)	7 (29.2)	NS
Ischemic heart disease	4 (11.1)	2 (8.3)	NS
Prior stroke	5 (13.9)	0	NS
Atrial fiblliration	9 (25)	0	0.022*
Chronic heart failure	9 (25)	0	0.022*
Chronic kidney disease	3 (8.3)	0	NS

Ages are expressed as the mean ± standard deviation. Other data are shown as absolute numbers, with percentage in parentheses. Statistical analysis was performed using Student’s t test or *Fisher’s exact probability test. NS = not significant.

**Table 2:** Clinical courses and complications in patients with ischemic stroke.

		Total patients	Patients with PI	Patients with SAI
Number		36	13	9
Clinical phase	Hyperacute (day 0-2)		7	4
	Acute (day 3-7)		6	5
	Early subacute (day 8-16)		0	0
	Late subacute/chronic (day 17~)		0	0
TOAST	LAA	14	6	4
classification	CE	10	5	5
	SAO	10	2	0
	SOC	1	0	0
	SUC	1	0	0
NIHSS score	1-10	27	7	3
on admission	11-20	5	3	3
	21-40	4	3	3

Each number represents the number of patients according to the classification.

PI: progressing infarction, SAI: stroke-associated infection, TOAST: Trial of Org 10172 in Acute Stroke Treatment, LAA: large artery atherosclerosis, CE: cardioembolism, SAO: small artery occlusion, SOC: stroke of other determined cause, SUC: stroke of undetermined cause, NIHSS: National Institute of Health Stroke Scale

### Dynamics of blood monocyte subsets after acute stroke

**Figure 2:**
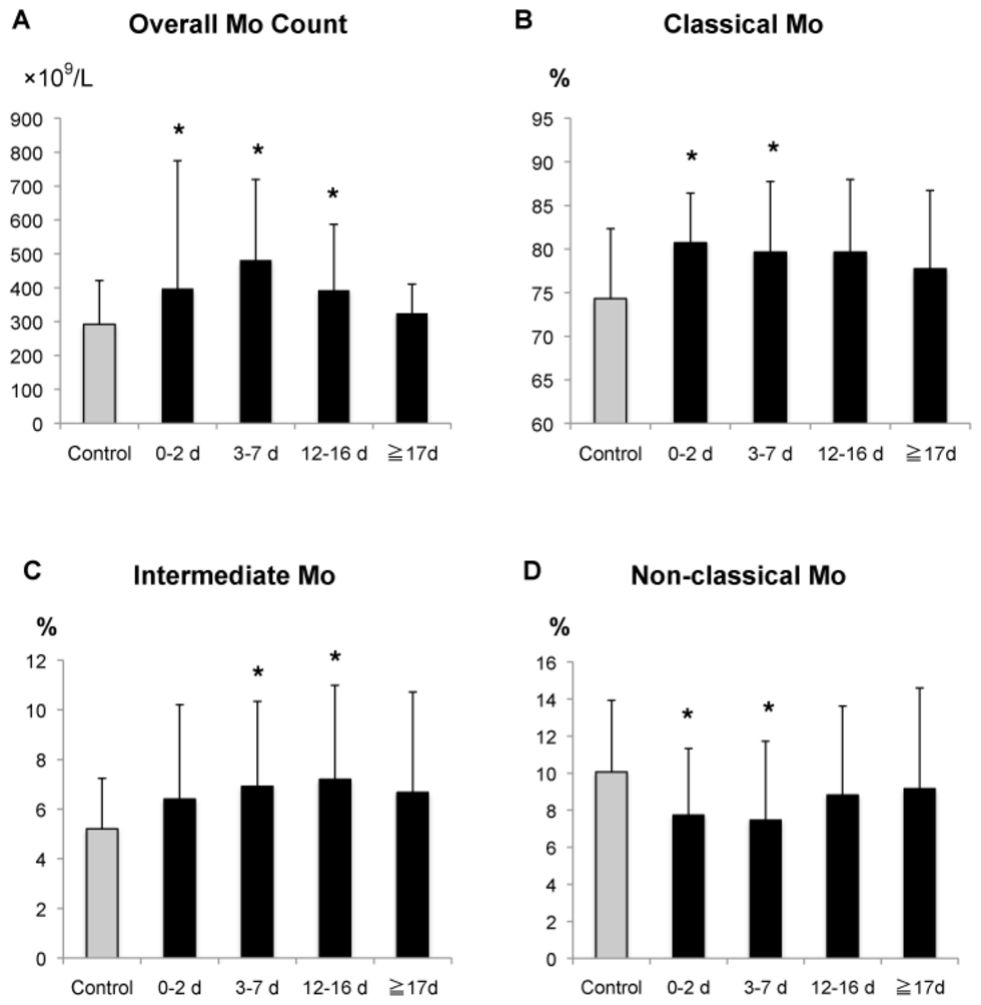
Time courses of monocyte subsets after stroke. (**A**) The overall number of circulating monocytes was increased. The percentages of (**B**) classical and (**C**) intermediate Mo were increased, whereas that of (**D**) non-classical Mo was decreased. Bars show the mean ± SEM. *p<0.05, t-test, compared to control. Control, n=24; day 0-2, n=36; day 3-7, n=35; day 12-16; n=33; day of hospital discharge, n=26.

Overall monocyte counts were significantly elevated during the 0-16 days after stroke as compared to the controls (Figure 2A). Classical Mo showed a significant increase in percentage from 0–7 days (Figure 2B) and intermediate Mo showed a significant increase from 3–16 days (Figure 2C) after stroke. In contrast, the percentage of non-classical Mo was significantly reduced from 0–7 days (Figure 2D).

### Correlations between monocyte subsets and NIHSS score on admission

**Figure 3:**
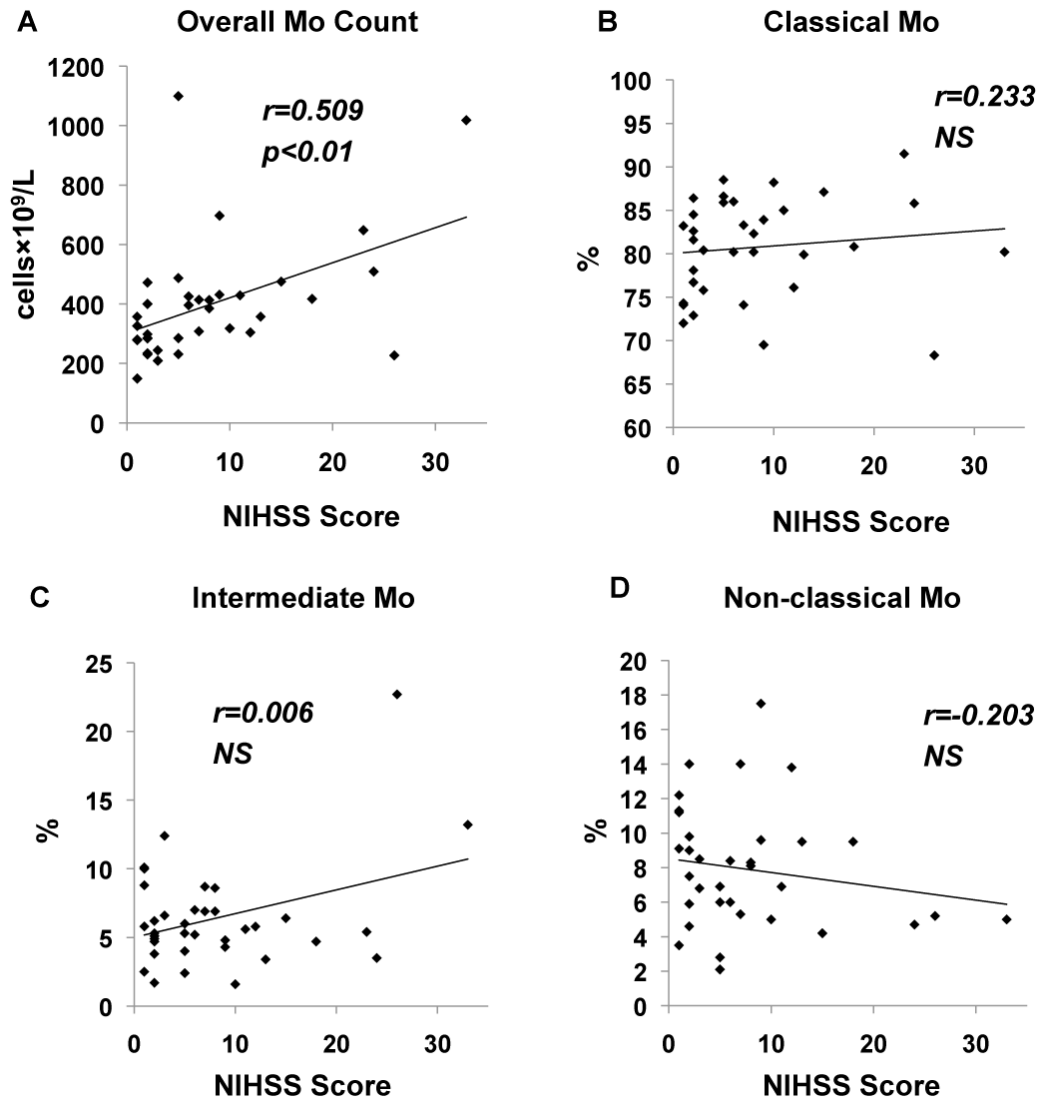
Correlation between monocytes and NIHSS score on admission. (**A**) There was a significant positive correlation between overall monocyte count and NIHSS score, (**B**–**D**) while none was seen between the monocyte subsets and that score.

There was a significant positive correlation between monocyte count and NIHSS score on admission (r=0.509, p<0.01; Spearman’s rank correlation coefficient), but not among the 3 monocyte subsets (Figure 3).

### Correlations between stroke subtype and NIHSS score on admission

NIHSS scores on admission were compared among patients with stroke due to LAA (n=14), CE (n=10), and SAO (n=10). There was no difference between the LAA and CE groups, whereas the scores in the SAO group were significantly lower than those in the LAA (p=0.013) and CE groups (p=0.017) when analyzed using a t-test (data not shown).

### Stroke subtype-specific changes in monocyte subsets

**Figure 4:**
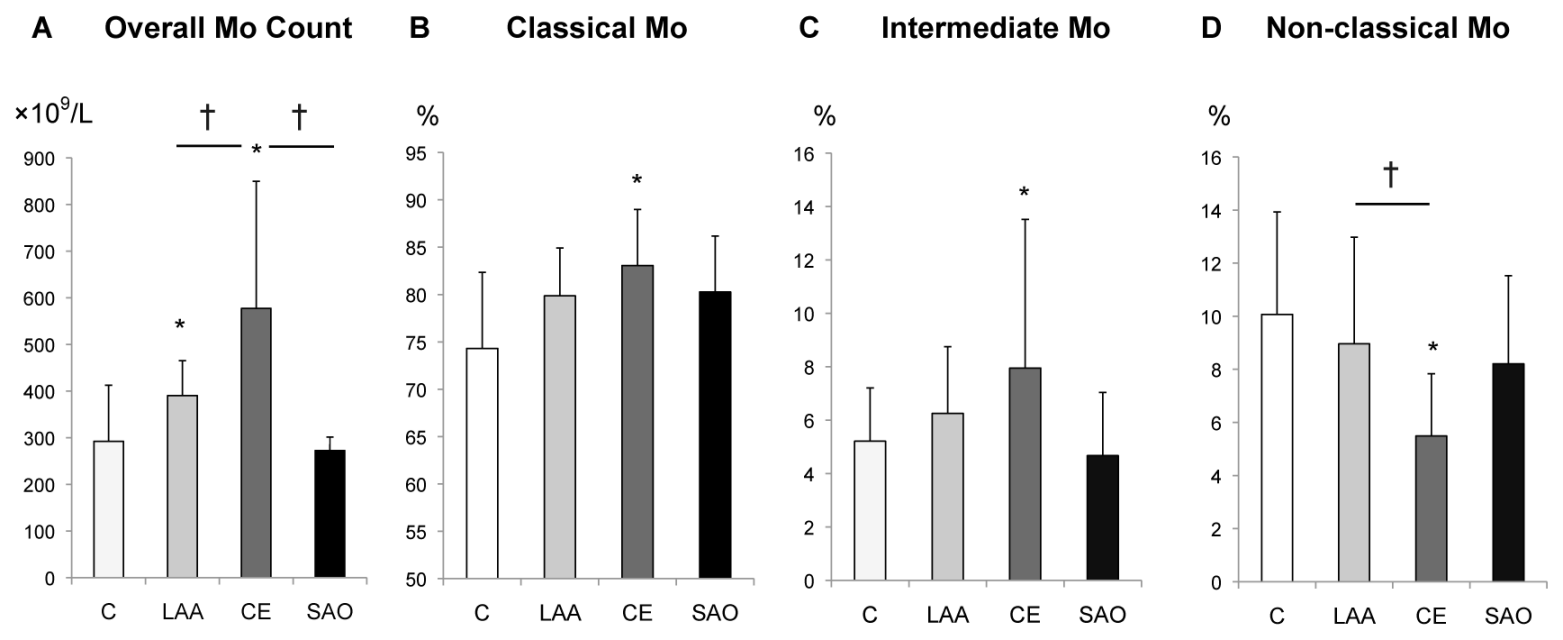
Stroke subtype and monocyte subsets from day 0-2 (hyperacute phase). (**A**) The overall monocyte counts in the LAA and CE groups were significantly increased as compared to the control. (**B**, **C**, **D**) The percentages for the monocyte subsets in the CE group were significantly changed as compared to the control. Bars show the mean ± SEM. *p<0.05, t-test, compared to control. †p<0.05, ANOVA, compared to CE. C: control; LAA: large artery atherosclerosis; CE: cardioembolism; SAO: small artery occlusion.

**Figure 5:**
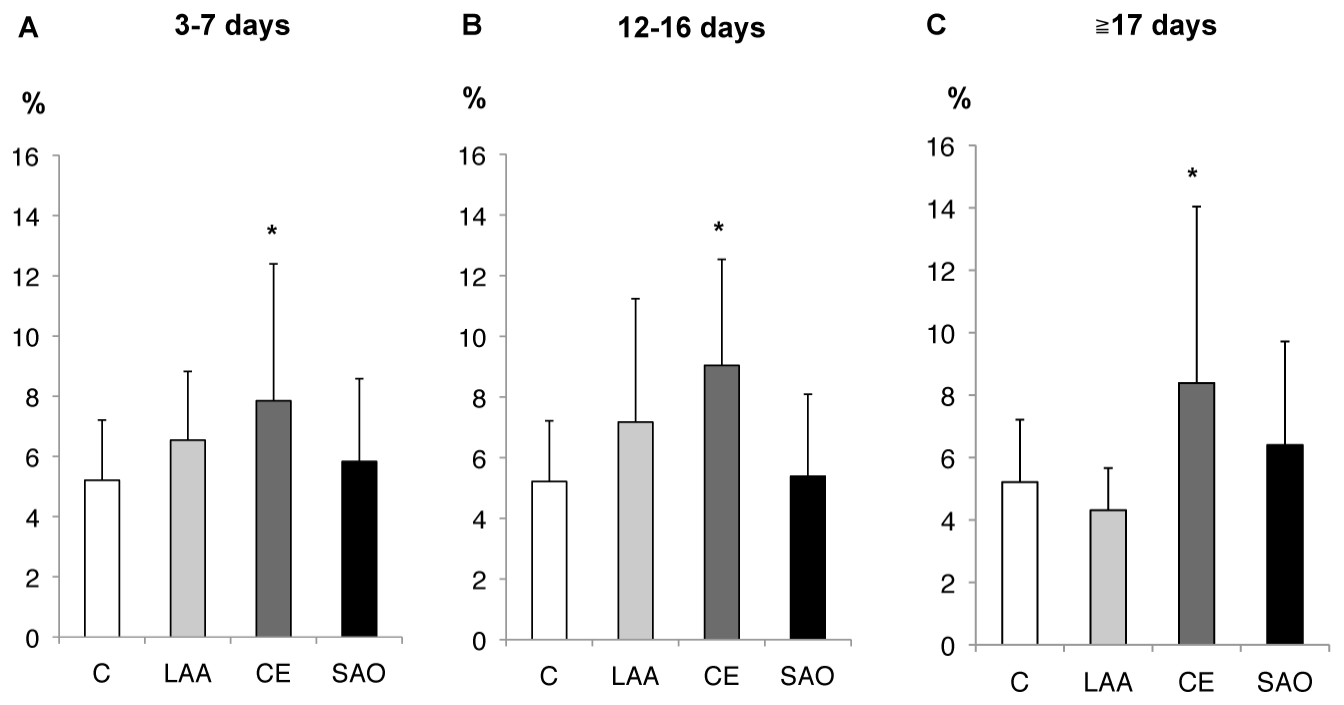
Stroke subtypes and intermediate monocytes after hyperacute phase of stroke. (**A**–**C**) A significantly elevated percentage of intermediate Mo in the CE group persisted until the day of discharge. *p<0.05, t-test, as compared to control. C: control; LAA: large artery atherosclerosis; CE: cardioembolism; SAO: small artery occlusion.

Overall monocyte counts in the LAA and CE groups were significantly increased as compared to the control group from 0–2 days after stroke (Figure 4A). Moreover, monocyte counts in the CE group were significantly higher than in the other stroke groups. The CE group was unique, as classical and intermediate Mo were increased, while the percentage of non-classical Mo was decreased during the hyperacute phase as compared to the control (Figure 4B–D). Furthermore, a significantly elevated percentage of intermediate Mo in the CE group persisted until discharge (Figure 5).

### Event-specific changes in monocyte subsets

**Figure 6:**
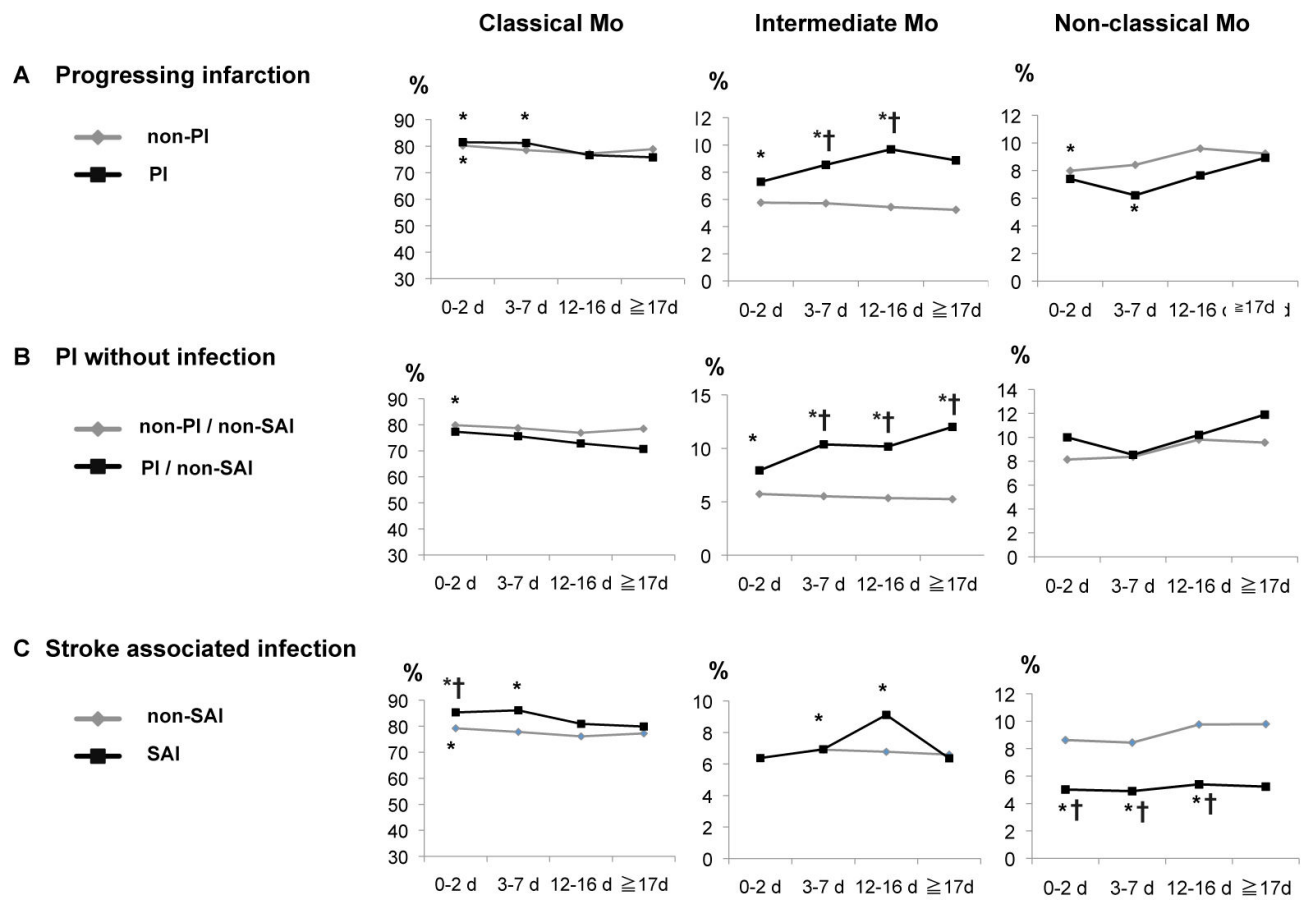
Time courses of monocyte subsets regarding stroke complications. Classical and intermediate Mo were increased, while non-classical Mo were decreased. Patients were divided into (**A**) with or without PI, (**B**) excluding SAI, (**C**) and with or without SAI. *p<0.05, t test, compared to control. †p<0.05, t test, compared to other group in category. PI = progressing infarction, SAI = stroke-associated infection.

The time courses of monocyte subsets in patients with PI and/or SAI are shown in Figure 6. In PI patients, the percentages of classical Mo were increased from 0–7 days after stroke, while those of intermediate Mo were increased from 0-16 days as compared to the controls (Figure 6A). Also, the percentage of intermediate Mo in the PI group was significantly higher from 3–16 days as compared to the non-PI group (Figure 6A). That finding was enhanced when patients with SAI were excluded from analysis, which revealed a persistent increase after stroke (Figure 6B). In SAI patients, the percentage of non-classical Mo was remarkably decreased from 0–16 days after stroke as compared to the control and non-SAI patients (Figure 6C). The SAI patients showed an increased percentage of classical Mo from 0–7 days and that of intermediate Mo from 3–16 days after stroke as compared to the controls (Figure 6C).

### Serum cytokine levels after stroke

**Figure 7:**
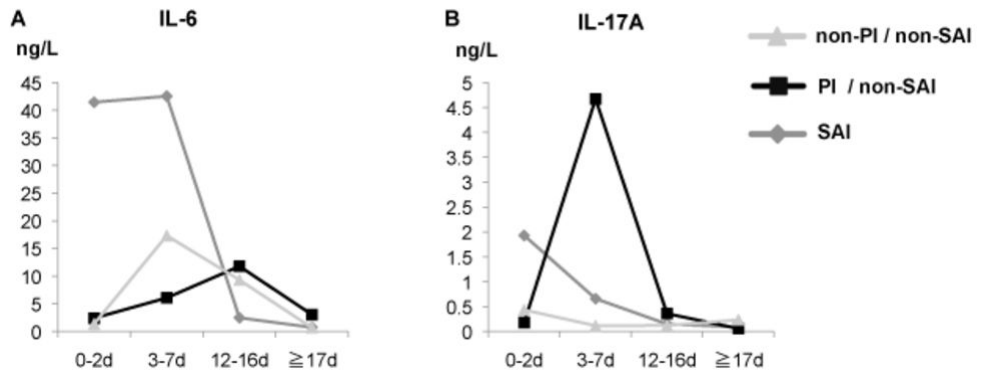
Dynamics of pro-inflammatory cytokines after stroke. (**A**) In the SAI group (closed diamond), serum IL-6 levels were elevated from 0–7 days after stroke, (**B**) while serum IL-17A levels were elevated in the PI group without SAI (closed square) from 3–7 days after stroke.

We measured a panel of cytokines using 144 serum samples obtained during different phases of stroke from the 36 stroke patients. Substantial elevations in the levels of IL-6 and IL-17A were observed in 44 (30.6%) and 28 (19.4%) samples, respectively. However, other cytokines including IL-1β, IL-10, TNF-α, and TGF-β were undetectable. Both IL-6 and IL-17A in serum reached a peak level during the acute phase (3-7 days) after stroke. The peak IL-6 level was most prominent in patients with SAI, while that of IL-17A was most apparent in PI patients without infection (Figure 7).

### Relationships of monocyte subsets with levels of IL-6 and IL-17A in non-SAI patients

**Figure 8:**
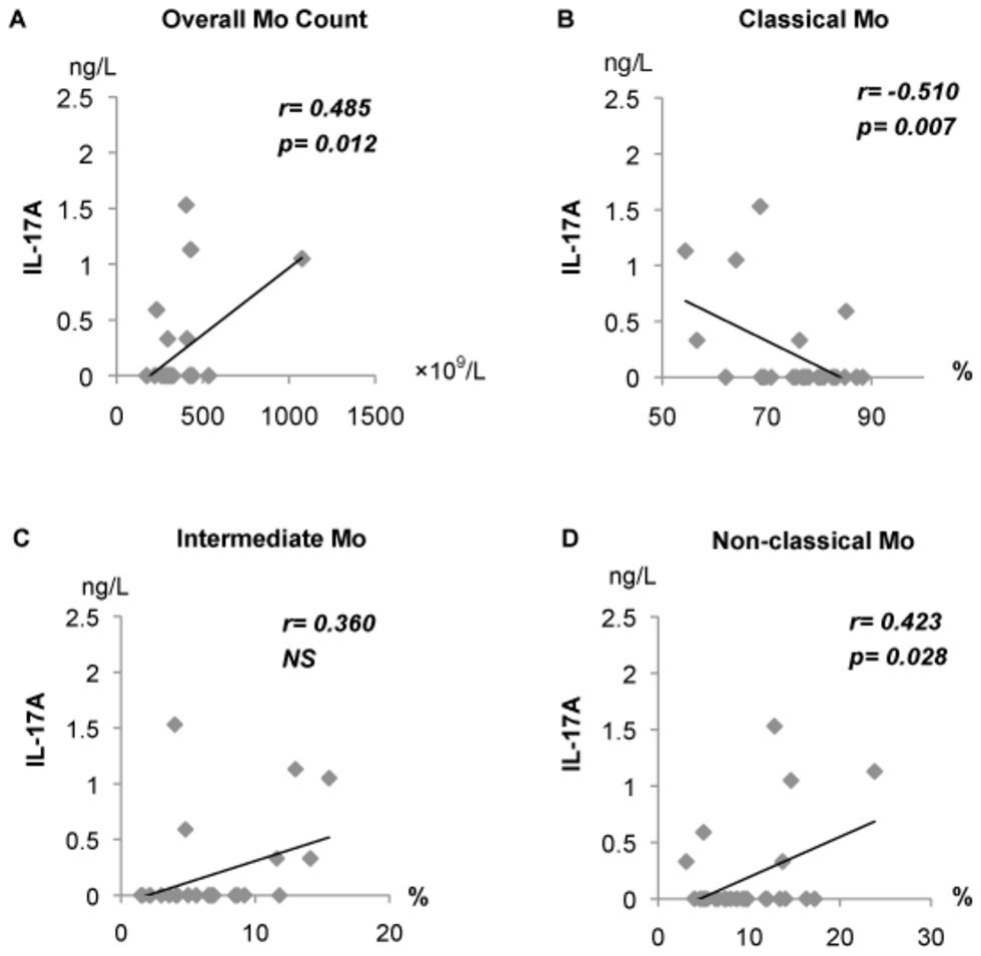
Relationship between monocyte subsets and serum IL-17A in early subacute phase (12-16 days) after stroke. IL-17A level was positively correlated with (**A**) monocyte count and (**D**) percentage of non-classical Mo, and negatively with (**B**) percentage of classical Mo. Statistical analysis was performed using Spearman’s rank correlation coefficient.

In order to elucidate factors that affect the clinical course of ischemic stroke, IL-6 and IL-17A levels were analyzed in patients not complicated with infection (non-SAI). From 0–2 days after stroke (hyperacute phase), IL-6 and IL-17A levels showed positive correlations with overall monocyte counts (r=0.556, p<0.001, and r=0.597, p=0.001, respectively; Spearman’s rank correlation coefficient, data not shown). However, from 3–7 days after stroke (acute phase) no correlations were found. In contrast, from 12–16 days after stroke (early subacute phase), IL-17A levels showed positive correlations with overall monocyte counts (r=0.485, p=0.012, Figure 8A) and the percentage of non-classical Mo (r=0.423, p=0.028, Figure 8D), and a negative correlation with the percentage of classical Mo (r=-0.510, p=0.007, Figure 8B), when analyzed using Spearman’s rank correlation coefficient. Furthermore, during the late subacute/chronic phase (≥17 days after stroke), IL-6 level was positively correlated with the percentage of intermediate Mo (r=0.661, p=0.001; Spearman’s rank correlation coefficient, data not shown).

## Discussion

The present findings showed that monocytes increased from 0–16 days after ischemic stroke in our patients, while the percentages of both classical and intermediate Mo were increased, and that of non-classical Mo was decreased during the acute phase. Our results agree with those reported by Urra et al. [14,22], except for their finding that the CD14^high^CD16^-^ classical Mo subtype was unchanged, which could be attributed to the monoclonal antibodies used in that phenotyping study. On the other hand, in an experimental stroke model, Ly6C^high^ inflammatory monocytes, which correspond to CD14^high^CD16^-^ classical Mo in humans, were found to be deployed from the spleen, and potentially contributed to brain inflammation and injury [13]. In addition, infarct volume shown by MRI was reported to be correlated with the proportion of CD14^high^CD16^-^ Mo [22]. Therefore, increased CD14^high^CD16^-^ Mo represents a population of monocytes rapidly recruited from the spleen to circulation, where they subsequently migrate to ischemic tissues, leading to further brain damage.

In regard to stroke subtype, we found that cardioembolic infarction impacted the dynamics of monocyte subsets in the present CE patients, as the percentage of intermediate Mo was persistently high until the day of discharge. This cannot simply be attributable to the severity of brain damage, as NIHSS scores were similar between the CE and LAA groups, and not correlated with any of the 3 monocyte subsets. Instead, this phenomenon may indicate difficultly for patients with CE to rapidly acquire ischemic tolerance, such as metabolic downregulation and angiogenesis, which is easily achieved during the chronic process of oligemia preceding atheromatous infarction (LAA) [23]. Ischemic tolerance has been shown to reduce infarct volume, BBB disruption, and leukocyte migration in experimental models [24]. Since patients with LAA and SAO infarction have likely been exposed to ischemic events more frequently than those with CE, ischemic tolerance may mask the altered dynamics of monocyte subsets in those stroke subtypes. Thus, CD14^high^CD16^+^ intermediate Mo, persistently increased in patients with CE, may play a pivotal role in ischemic pathophysiology.

We also found that the CD14^high^CD16^+^ intermediate Mo subset was persistently increased in patients who exhibited worsening infarction (PI). Since other investigators have reported increased CD14^high^CD16^+^ monocytes in patients with SAI [22], we also analyzed this population by comparing PI patients with and without SAI. There was no difference seen, demonstrating the relevance of CD14^high^CD16^+^ intermediate Mo to PI. This finding can be interpreted as a protective response of the immune system to minimize inflammatory damage in the brain [22], since intermediate Mo were reported to be the main producers of anti-inflammatory IL-10 [25]. However, it is also known that those cells possess high phagocytic activity and an antigen-presenting function [25]. In addition, human studies have shown that CD14^high^CD16^+^ monocytes are expanded in chronic inflammatory disease processes including coronary artery disease [26] and rheumatoid arthritis [27]. Together, these findings suggest that CD14^high^CD16^+^ monocytes have a potential pro-inflammatory function. Furthermore, CD14^+^CD16^+^ monocytes were shown to increase the expression of inducible nitric oxide synthase (iNOS) [28], which participates in the mechanism of cerebral ischemic injury [29]. Therefore, intermediate Mo might have been involved in expansion of ischemic tissue damage in our PI patients.

The percentage of the CD14 ^dim^CD16^+^ non-classical Mo subset was decreased during the hyperacute and early subacute phases in SAI patients. This may have been due to rapid migration of those cells into inflammatory tissue, as previous reports have noted that CD14 ^dim^CD16^+^ monocytes have a higher migratory potential than other monocyte subsets [30]. In this regard, Ly6C^low^ non-classical monocytes in mice, which are homologous to CD14 ^dim^CD16^+^ monocytes in humans, have been reported to patrol in search of signs of infection or tissue damage, and rapidly exit vessels to reach the inflammation site within several hours after infection [31]. Conversely, a reduction in non-classical Mo may be associated with monocyte deactivation usually seen after stroke [14,32], which in turn could render patients susceptible to bacterial infection (SAI state). Although an increased proportion of CD14 ^dim^CD16^+^ monocytes was reported to predict better outcome following stroke [22], we found no association with progressing infarction. Collectively, the CD14 ^dim^CD16^+^ non-classical Mo subset appears to be more closely associated with inflammation induced by infection as compared to that related to cerebral ischemia.

Finally, the elevated serum IL-6 and IL-17A levels observed in the present study deserve mention. That of IL-6 seemed to reflect the status of complicated infection, as it reached a peak from 0–7 days after stroke and rapidly decreased to an unremarkable level in patients with SAI. In contrast, IL-17A may be associated with pathological processes occurring in the ischemic brain. IL-23 produced by activated macrophages induces IL-17 production from γδT cells and causes further ischemic damage during the delayed phase of brain ischemia [33], while CD14^high^CD16^+^ intermediate Mo were reported to promote Th17 responses in rheumatoid arthritis [34]. In the present study, we found that CD14 ^dim^CD16^high^ non-classical Mo had a positive correlation with serum IL-17A from 12–16 days after stroke, suggesting that CD16^+^ monocytes producing IL-17 have a role in the pathogenesis of brain ischemia after the acute phase. Further studies will be of value to more precisely clarify the roles of intermediate and non-classical Mo, both of which express various levels of CD16 molecules, in regard to overall clinical outcome of stroke patients and IL-17 production.

## Conclusions

Distinct monocyte subsets are associated with complications during acute and subacute phases of ischemic stroke. CD14^high^CD16^+^ intermediate Mo may be involved in CNS tissue damage in progressing infarction, while CD14 ^dim^CD16^high^ non-classical monocytes were shown to be relevant to stroke-associated infection. However, there are limitations to the present results that must be considered. Since the study was observational, it is not firmly conclusive whether our findings were causative or a result of PI and SAI. Therefore, two types of future investigations are necessary; a follow-up study of post-stroke patients for recurrent infarction and a cohort study of healthy elderly subjects afflicted by ischemic stroke, both of which should focus on CD14^high^CD16^+^ intermediate and CD14 ^dim^CD16^high^ non-classical monocytes. Such approaches may pave the way for development of a new treatment strategy for acute ischemic stroke.
